# Complications and long-term outcomes after open surgery for traumatic subaxial cervical spine fractures: a consecutive series of 303 patients

**DOI:** 10.1186/s12893-016-0172-z

**Published:** 2016-08-15

**Authors:** Hege Linnerud Fredø, Syed Ali Mujtaba Rizvi, Mehran Rezai, Pål Rønning, Bjarne Lied, Eirik Helseth

**Affiliations:** 1 Faculty of Medicine, University of Oslo, Oslo, Norway; 2 Department of Neurosurgery, Oslo University Hospital - Ullevål, N - 0407 Oslo, Norway; 3 Department of Neuroradiology, Oslo University Hospital, Oslo, Norway

**Keywords:** Spinal fractures, Cervical vertebrae, Subaxial, Injuries, Surgery, Complications, Mortality, Outcome

## Abstract

**Background:**

Patient selection for surgical treatment of subaxial cervical spine fractures (S-CS-fx) may be challenging and is dependent on fracture morphology, the integrity of the discoligamentous complex, neurological status, comorbidity, risks of surgery and the expected long-term outcomes. The purpose of this study is to evaluate complications and long-term outcomes in a consecutive series of 303 patients with S-CS-fx treated with open surgical fixation.

**Methods:**

Medical charts were retrospectively reviewed. The surviving patients participated in a prospective long-term follow-up, including clinical history, physical examination and updated cervical CT. Patients with ankylosing spondylitis were excluded from this study.

**Results:**

The median patient age was 48 years (range 14.7–93.9), and 74 % were males. Preoperatively, 43 % had spinal cord injury (SCI), and 27 % exhibited isolated radiculopathy. The median time from injury to surgery was 2 days (range 0–136). The risks of SCI deterioration and new-onset radiculopathy after surgery were 2.0 % and 1.3 %, respectively. Surgical mortality (death within 30 days after surgery) was 2.3 %. The reoperation rate was 7.3 %. At the long-term follow-up conducted a median of 2.6 years after trauma (range 0.5–9.1), 256 (99.2 %) of the patients who had survived and were living in Norway participated. Of the patients with American Injury Severity Scale (AIS) A–D at presentation, 51 % had improved one or more AIS grades. At the time of follow-up, 89 % of the patients with preoperative radiculopathy were without symptoms. Furthermore, 11 % of the patients reported severe neck stiffness, 5 % reported severe neck pain (Visual Analog Scale (VAS) ≥7), 6 % reported hoarseness, and 9 % reported dysphagia at the follow-up. The stable fusion rate, as evaluated using cervical-CT, was 98 %.

**Conclusions:**

In this large consecutive series of patients with S-CS-fx treated with open surgical fixation, the surgical mortality was 2.3 %, the risk of neurological deterioration was 3.3 % and the reoperation rate (any cause) was 7.3 %. The neurological long-term results were good, with 51 % improvement in AIS grade and resolution of radiculopathy in 89 % of the patients. Stable fusion was excellent and was achieved in 98 % of the follow-up group.

## Background

The incidence of traumatic cervical spine fractures (CS-fx) in the Norwegian population is estimated to be 15/100,000/year [1]. More than two-thirds of these cases (68 %) are subaxial CS-fx (S-CS-fx) [2]. Of the patients with S-CS-fx, 34 % are treated with open surgical fixation.

Surgical fixation may be performed using an anterior approach, a posterior approach, or a combined anterior/posterior (360° fixation) procedure. Indications for open surgical fixation of cervical fractures include instability, malalignment, and/or compression of the spinal cord or cervical nerve roots [3]. Other factors important for decision making are comorbidity, risks of surgery, and expected long-term outcomes after surgical fixation and/or external fixation [4–15]. Thus far, relatively few large series have been published on complications and long-term outcomes [6, 7, 11].

In this large, consecutive, contemporary series, we present surgery-related complications and long-term clinical outcomes after surgical fixation of S-CS-fx. Patients with ankylosing spondylitis were excluded from this series, and will be published in a separate paper.

## Methods

### Hospital

Oslo University Hospital, Ullevål (OUS-U), is the largest trauma center in Norway with a defined catchment area of 2.7 million people in 2010 [16]. The neurosurgical department at OUS-U is the only department performing open fixations for traumatic cervical spine fractures within this patient population.

### Inclusion criteria

- Trauma patients admitted to OUS-U between January 2002 and December 2010
- Diagnosed subaxial (C3–C7) cervical spine fracture (S-CS-fx) on cervical CT with 2-D reconstruction in 3 planes
- S-CS-fx treated with open fixation surgery

### Exclusion criteria

- S-CS-fx treated non-surgically
- Ankylosing spondylitis
- Neoplastic fractures

### Variables registered based on chart review

The following variables were recorded based on a retrospective chart review: sex, age, trauma mechanism according to ICD-10 [17], Head Injury Severity Scale (HISS) [18], spinal cord injury (SCI), preoperative American Spinal Injury Association impairment scale (AIS) [19], preoperative radiculopathy (only registered if the patient did not have a SCI (AIS E)) (yes/no), primary intention to treat (surgery/conservative treatment converted to surgery), surgical method (anterior cervical decompression and fusion (ACDF) with anterior plating, corpectomy with autologous iliac crest graft and anterior plating, posterior fusion (wiring or lateral mass screws) with or without cervical laminectomy, and combined anterior-posterior fusion (360° fusion)), time from injury to surgery, postoperative AIS and radiculopathy (yes/no), surgery for postoperative hematoma (yes/no), surgery for postoperative infection (yes/no), surgery for postoperative CSF leakage (yes/no), reoperation (suboptimal reposition, suboptimal placement of fixation material, breakage of fixation material, pseudarthrosis, secondary loss of alignment, or suboptimal decompression), and surgical mortality within 30 days.

The fractures were retrospectively scored according to the Subaxial Injury Classification system (SLIC) published by Vaccaro et al. in 2007 [3]. This classification of S-CS-fx is based on the fracture morphology, discoligamentous integrity and neurological status. Preoperative radiological examinations were evaluated by an experienced neurosurgeon and a neuroradiologist. Both assigned SLIC points for morphology and discoligamentous injury at each subaxial injured level. In circumstances in which the points assigned did not coincide, the radiological examinations were jointly studied for consensus. The points for neurological status were assigned based on the patient chart reviews. In patients with multiple injured levels, the highest SLIC score was registered. Treatment recommendations based on the SLIC scores were non-surgical if <4 points, equivocal if =4 points, and surgical if >4 points; however, a number of concomitant factors must be considered in addition to the score.

### Surgery and postoperative management

In each case, the choice of surgical method was determined by the treating surgeon. A broad spectrum of S-CS-fx injury patterns exists, and no consensus or scientific evidence provides definitive guidelines regarding the surgical approach. More than one surgical technique can accomplish the same goals. The basic surgical goals are adequate decompression of the neural elements, restoration of alignment, and sufficient mechanical spinal stability.

In our department, the following techniques were used: 1. Anterior cervical decompression and fusion (ACDF) with anterior plating (CSLP® or Vectra®, both from Synthes, Solothurn, Switzerland), which in the earlier years of the study was performed with an autologous iliac crest graft, and in the subsequent years was replaced with an artificial polyetheretherketone (PEEK) cage (Cervios chronOS®, Synthes, Solothurn, Switzerland); 2. Corpectomy with autologous iliac crest graft and anterior plating; and 3. Posterior fixation, which during the initial years of the study was mainly performed using cerclage wiring techniques (0.8 mm stainless steel surgical wire) and was then subsequently replaced by lateral mass screw fixation with rods (Axon® and Synapse®, both Synthes, Solothurn, Switzerland), with or without decompressive laminectomy. When the posterior screw fixation required extension beyond the cervicothoracic junction, the lower screws were placed in the thoracic pedicles. In cases where neither an anterior nor a posterior approach alone offered sufficient mechanical stability, we used a combination of anterior and posterior fixation (360° fixation). In cervical anterior approaches we used microscope after the soft-tissue approach were established. In posterior approaches, microscope was not routinely used. We did not have access to perioperative neuromonitoring, nor spinal navigation.

For perioperative antibiotic prophylaxis, we routinely used a single dose of i.v. cephalothin (2 g) 15–30 min before the skin incision, which was repeated after 180 min in cases of ongoing surgery. Alternatively, i.v. clindamycin (600 mg) was used in cases of penicillin-allergy. Thrombosis prophylaxis included compression stockings and early mobilization. Low-molecular-weight heparin was administered to patients who could not be mobilized within 24 h. Most patients received a stiff collar for 12 weeks postoperatively.

### Long-term prospective clinical follow-up

Vital status (dead or alive) and time of death were obtained from the Norwegian Population Registry (Folkeregisteret) on the 20^th^ of June 2012. At this time, 258 patients were alive and living in Norway, 25 had expired, and 20 were not found in the Norwegian Population Registry (they were living outside of Norway and were not available for follow-up).

Of the 258 patients living in Norway, two patients rejected the invitation to participate in the follow-up examinations. Thus, 256/258 (99 %) of the surviving Norwegian patients participated in the follow-up evaluations during 2010–2012 (clinical examination and cervical CT).

The following variables were recorded: time from injury to clinical follow-up (months), neurological status by AIS and radiculopathy presence (yes/no), neck pain using the Visual Analog Scale (VAS) [20], self-reported neck stiffness (none, mild, severe), hoarseness (yes/no) and swallowing difficulties (yes/no). The following variables were registered based on the follow-up CTs: bony healing of fracture lines (certain/uncertain), altered position/fracture of the fixation material/graft (yes/no), and secondary loss of alignment (yes/no). We defined stable fusion as healed fracture lines, lasting alignment, and no dislocation or failure of hardware at time of long-term follow-up.

### Ethics

The study was approved by the data protection official at Oslo University Hospital. 

### Database and statistical analysis

The data were described using counts, percentages, median, means, ranges and standard deviations when appropriate. Overall survival (OS) analyses were performed using Kaplan-Meier curves, which measured survival from the time from surgery to the time of death. Furthermore, a Cox regression model was fit to investigate the effects of age, AIS grade and head injury on survival. Due to the few events, we dichotomized the head injury variable for this analysis into HISS 0–2 (none, minimal and mild) and HISS 3–4 (moderate and severe). A preoperative AIS grade was not assigned to 15 patients because of head-injury and intubated status. By performing a complete case analysis, we lost statistical power and obtain biased results. Thus, we performed a multiple imputation procedure. This procedure ensures that multiple complete datasets are generated where the missing variables are varied based on the observed values for the individual and the relationship observed in the dataset for the other participants. Because the missing values were replaced by multiple values in the different datasets, the subsequent analysis also reflected the uncertainty in the value generated, resulting in accurate standard errors. We used MICE for this purpose and generated ten datasets [21].

For ordinal responses on the VAS scale and neck stiffness, ordinal regression was performed to investigate any associations between the ordinal variable and the surgical approach. Linear contrasts between the different surgical approaches were analyzed from the ordinal model.

A *p*-value <0.05 was considered statistically significant. Statistical analyses were performed using SPSS v16.0 (SPSS Inc., Chicago, IL, USA) and R v 3.2 [18].

## Results

### Patient characteristics

In total, 303 consecutive patients with S-CS-fx treated with open surgical fixation were included in this study. The patient characteristics are provided in Table 1. There were 225 (74 %) males, and the median and mean age at the time of injury were 48 and 49 years, respectively (range 15–93 years). At the time of surgery, 129/303 (43 %) had evident SCI (AIS A–D). Radiculopathy was only registered if the patient did not have a SCI. Radiculopathy as the only neurological symptom was present in 81/303 patients (27 %). Fifteen patients (5 %) were unconscious and could not be assessed for neurological status. The mean SLIC score at the time of surgery was 6.5 (range 2–10).

**Table 1:** Patient characteristics (*n* = 303)

	*N* (%)
Sex	
Male	225 (74)
Female	78 (26)
Age (years)	
≤19	24 (8)
20–39	88 (29)
40–59	96 (32)
60–79	67 (22)
≥80	28 (9)
Injury mechanism	
Falls	156 (51)
Motorized vehicle accident	86 (28)
Pedal cyclist injured in transportation accidents	19 (6)
Pedestrian injured in transportation	6 (2)
Assault	5 (2)
Diving	14 (5)
Others	17 (6)
Head injury severity scale	
None	42 (14)
Minimal	119 (39)
Mild	106 (35)
Moderate	22 (7)
Severe	14 (5)
ASIA impairment scale preoperatively	
A (complete)	45 (15)
B (incomplete; sensory but no motor function below neurological level, including sacral segments)	17 (6)
C (incomplete; motor function preserved below neurological level with more than half of the key muscles graded <3/5)	16 (5)
D (incomplete; motor function preserved below neurological level with more than half of the key muscles graded ≥3/5)	51 (17)
E (normal)	159 (52)
Not possible to grade due to head injury/multitrauma	15 (5)
Type of surgery	
ACDF with anterior plating	155 (51)
Corpectomy with autologous iliac crest graft and anterior plating	54 (18)
Posterior fusion with wiring technique	42 (14)
Posterior fusion with lateral mass screws	24 (8)
Combined anterior-posterior fusion (360° fusion)	28 (9)
SLIC points at time of first surgery	
1	0
2	8 (2.6)
3	15 (5.0)
4	32 (10.6)
5	43 (14.2)
6	58 (19.2)
7	50 (16.5)
8	31 (10.2)
9	38 (12.5)
10	28 (9.2)

### Surgical treatment

The primary intention to treat with surgery was assigned in 269 patients (89 %) (group A), whereas 34 patients were reassigned from conservative to surgical treatment (11 %) (group B). In group A, the median time from trauma to open surgical fixation was 2 days (range 0–80 days), 12/269 (5 %) patients were operated within 12 h after trauma, 56/269 (21 %) patients were operated 12–24 h after trauma, and a total of 174/269 (65 %) patients were operated within 3 days after their injury (Table 2). Of the 26 patients in group A who were operated more than 10 days after injury, 17 patients had a delayed diagnosis, four patients were Norwegians who were injured abroad, and the remaining five patients had life-threatening injuries to other organ systems. In group B, the median time to surgery was 30 days (range 2–136). The types of surgery are shown in Table 1.

**Table 2:** Time from injury to surgery

Time to surgery	Number of patients (%) Group A^a^	Number of patients (%) Group B^b^
<12 h	12 (5)	
12–24 h	56 (21)	
1 day	36 (13)	
2 days	42 (16)	1 (2.9)
3 days	28 (10)	
4 days	20 (7)	1 (2.9)
5 days	13 (5)	2 (5.9)
6 days	13 (5)	2 (5.9)
7 days	7 (3)	2 (5.9)
8 days	8 (3)	2 (5.9)
9 days	6 (2)	1 (2.9)
10 days	2 (<1)	
11 days	3 (1)	
12 days	2 (<1)	2 (5.9)
13 days	3 (1)	
Third week	6 (2)	2 (5.9)
Fourth week	6 (2)	1 (2.9)
Second month	5 (2)	8 (23.6)
Third month or later	1 (<1)	10 (29.4)
Total	269 (100)	34 (100)

^a^Group A: Primary intention to treat was surgical

^b^Group B: Reassigned from conservative treatment to surgery

Compliance to SLIC treatment recommendations solely based on the assigned points (≥4 points) was 92.4 %. A total of 23 patients (7.6 %) with less than four SLIC points were surgically treated (Table 1).

### Neurological deterioration after surgery

SCI deterioration after surgery was detected in 6/303 (2.0 %) patients (Table 3). In three of our patients, we could not detect any specific reason for the deterioration, other than the probable inherent vulnerability of these patients to intubation, surgical positioning, and mechanical forces during surgery. Deterioration was caused by a postoperative hematoma in one patient, by a deep wound infection in one patient, and because the lateral mass screws were placed too medially in one patient.

**Table 3:** SCI deterioration secondary to open fixation surgery

No	Sex	Age	Surgical approach	AIS grade			
				Preop	Postop	Discharge	Follow-up
1	M	17	Anterior	D	C	D	D
2	M	42	Posterior	D	D^a^	D	D
3	F	57	Posterior	E	D	D	E
4	M	59	Anterior	C	A	A	D
5	M	87	360°	C	A	A	^b^
6	M	86	Anterior	D	A	^b^	^b^

^a^Worsened paresis within the same AIS grade

^b^Expired within 30 days after surgery

Four of the 303 patients (1.3 %) had new-onset radiculopathy after surgery, three patients were operated using anterior approach and one patient was operated using posterior approach.

### Second and third surgeries due to surgical complications/failures

A second surgery due to surgery-related complications/failures after primary fixation was performed in 22/303 (7.3 %) patients (Table 4). The indications for a second surgery were malalignment in eight patients, infection in four patients, suboptimal placement of the screws/plates in three patients, fixation device failure in four patients, suboptimal decompression in two patients, postoperative CSF leakage in two patients, deterioration of SCI after the first surgery in three patients, and postoperative hematoma in one patient (the sum of the indications was 27 because some patients had more than one indication for the second surgery). Of the 22 patients undergoing a second surgery, four patients required a third surgery due to complications after the second surgery (Table 4).

**Table 4:** Second surgery due to surgery-related complications/failures after primary fixation was performed in 22/303 patients

No	Sex	Age	Surg.tech/levels primary surgery	Indication for second surgery	Timing of second surgery (days)	Complication to second surgery	Third surgery
1	M	37	ACDF C6/7	Malalignment	8	None	No
2	M	26	Corpectomy C4-C6	CSF leakage	18	None	No
3	M	38	Corpectomy C5-C7	Fix.device failure, Malalignment	9	Fix.device failure, malalignment	Yes
4	M	59	Corpectomy C3-C5	SCI worsening, not significantly decompressed, fix.device failure	4	None	No
5	M	15	Cerclage C6/7	Malalignment	156	None	No
6	M	19	Corpectomy C4-C6	Suboptimal placement screws/plate	4	None	No
7	F	76	Corpectomy C6-Th1	Malalignment	18	None	No
8	M	70	360°; ACDF + post screws C5-Th2	Infection	14	None	No
9	M	60	ACDF C7/Th1	Fix.device failure	4	None	No
10	M	64	ACDF C7/Th1	Fix.device failure	2	Infection	Yes
11	F	80	360°; ACDF + post screws C4-Th1	Infection	15	None	No
12	F	32	ACDF C6/7	CSF leakage	165	CSF leakage	Yes
13	M	17	Corpectomy C4-C6	SCI worsening, postop hematoma	1	None	No
14	M	23	Corpectomy C4-C6	Malalignment	31	None	No
15	M	87	360°: ACDF + cerclage C5/6	SCI worsening, suboptimal placement screws/plate	1	None	No
16	F	74	ACDF C6/7	Suboptimal placement screws/plate	1	None	No
17	F	66	ACDF C4/5	Not significantly decompressed	3	None	No
18	M	67	Post screws C3-C7	Infection	7	None	No
19	M	64	ACDF C4/5	Malalignment	18	None	No
20	M	61	ACDF C6/7	Malalignment	7	None	No
21	M	74	Post screws C4-Th2	Infection	18	Infection	Yes
22	F	83	ACDF C5/6	Malalignment	39	None	No

### Surgical mortality and survival

Surgical mortality, which was defined as death within 30 days after surgery, occurred in 7/303 patients (2.3 %). Details of the fatalities are provided in Table 5.

**Table 5:** Patients who expired within 30 days of surgery

No	Sex	Age	Preop AIS grade	Postop AIS grade	HISS	MT	Type of surgery	Surgery-death (days)	Cause of death
1	M	86	D	A	Mild	Yes	Anterior	10	Respiratory failure
2	M	63	NT	NT	Severe	Yes	Anterior	2	Brain injury
3	M	84	D	D	Mild	Yes	Anterior	3	Respiratory failure
4	M	48	A	A	Severe	Yes	Anterior	4	Brain injury
5	M	82	A	A	Mild	Yes	360°	4	Respiratory failure
6	M	80	A	A	Minimal	No	Posterior	6	Respiratory failure
7	M	87	C	A	Moderate	No	360°	19	Respiratory failure

*MT* multitrauma, *NT* not testable

**Fig. 1:**
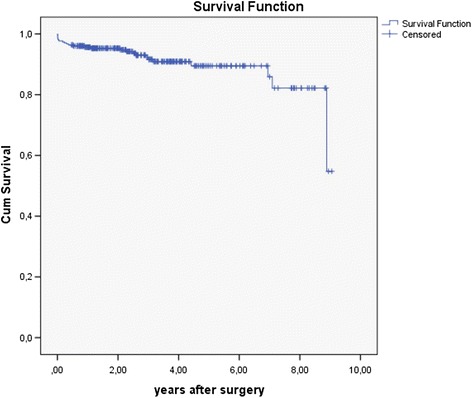
Kaplan-Meier: Survival after surgery

Twenty patients were living outside of Norway and were not found in the Norwegian Population Registry at the time of follow-up. These patients were excluded from the survival studies. The 1-, 2- and 5-year survival rates were 95.3, 94.9 and 88.3 %, respectively (Fig. 1). The mean age of the deceased patients was 71 years (range 33–87 years). Advanced age, severe head injury and severe SCI were significant negative prognostic factors with respect to overall survival (Table 6).

**Table 6:** Prognostic factors of overall survival

	HR	95 % CI	*p*-value
Age	1.09	1.06, 1.13	<0.001
HISS			
3–4	ref		
1–2	0.11	0.02, 0.57	0.008
AIS presurgery			
A	ref		
B	0.19	0.02, 1.69	0.137
C	0.16	0.02, 1.30	0.087
D	0.22	0.06, 0.75	0.015
E	0.18	0.06, 0.55	0.023

*HR* hazard ratio, *CI* confidence interval, *Ref* reference category

### Long-term follow-up examination

By the time of the long-term follow-up evaluation, 258/303 (85.1 %) patients were alive and living in Norway, 25/303 patients (8.3 %) had expired, and 20/303 patients (6.6 %) were not found in the Norwegian Population Registry (they were living outside of Norway and were not available for follow-up). Of the 258 living Norwegian patients invited to participate in the follow-up study, two patients refused participation, resulting in a follow-up rate of 99.2 % (256/258). The median and mean follow-up period was 2.6 and 3.1 years, respectively (range 0.5–9.0 years).

#### Neurological function

A comparison of the AIS grade before surgery and at the time of follow-up is provided in Table 7. None of the patients available for the follow-up had a lower AIS grade compared with the preoperative grade. Of the 103 patients who had evident SCI preoperatively and were available for the long-term follow-up, 41 patients were operated within 24 h after trauma (early group) and 62 patients after 24 h. In the early group, 48.8 % of the patients showed improved AIS grades, and 19.5 % improved ≥2 AIS grades. In the group operated after 24 h, 53.1 % of the patients showed improved AIS grades, and 12.9 % improved ≥2 grades. The improvement in AIS grades between the two groups were not significantly different (*p* = 0.442). Of the 75 patients with preoperative radiculopathy who were available for follow-up, only eight patients (11 %) had continued radicular symptoms. Of the four patients who had new-onset radiculopathy after surgery, three were asymptomatic at the follow-up.

**Table 7:** Comparison of pre-surgery and long-term follow-up AIS grade

Preoperative AIS		Follow-up AIS						
		A	B	C	D	E	Dead	Abroad/rejected follow-up
A	45	26	3	2	1	0	7	6
B	17	0	3	2	9	2	1	0
C	16	0	0	0	12	2	2	0
D	51	0	0	0	21	20	6	4
E	159	0	0	0	0	140	8	11
NT^a^	15	7	0	0	1	5	1	1
Totals	303	33	6	4	44	169	25	22

^a^ *NT* not testable preoperatively

#### Neck pain

The median VAS score for neck pain was 1 (range 0–10). Furthermore, 205/255 (80 %) patients had VAS scores ≤3, 37/255 (15 %) had VAS scores 4–6, and 13/255 (5 %) had VAS scores ≥7. One patient could not respond because of sequelae after head injury. No significant association was observed between the surgical approach and neck pain (posterior vs. anterior approach (*p* = 0.61), anterior approach vs. 360° approach (*p* = 0.78) and posterior vs. 360° approach (*p* = 0.50)).

#### Neck stiffness

Among the patients in this study, 67/255(26 %) reported no neck stiffness, 159/255 (63 %) reported mild neck stiffness and 29/255 (11 %) reported severe neck stiffness. One patient was unable to self-report on the issue. The patients who were fused with a posterior screw fixation reported significantly more neck stiffness than patients fused with an anterior approach alone (OR 2.28, 95 % CI (1.2–4.3), *p* = 0.01). We could not identify any significant difference between an anterior and a 360° approach.

*Sustained hoarseness* after surgery was reported by 15/255 patients (6 %). No difference in this complaint was observed with regard to the surgical approach (posterior vs. anterior (*p* = 0.7), anterior vs. 360° (*p*=0.2) and posterior vs. 360° (*p* = 0.7)).

#### Dysphagia

The number of patients reporting dysphagia at follow-up was 23/255 (9 %). Dysphagia after surgery was not significantly associated with any of the approaches (posterior vs. anterior (*p* = 0.16), anterior vs. 360° (*p* = 0.9) and posterior vs. 360° (*p* = 0.4)).

#### Radiological follow-up

Of the 256 patients available for follow-up with cervical CT scans, 252/256 (98.4 %) had a stable fusion, 1/256 (0.4 %) had a secondary loss of alignment, and 3 (1.2 %) had a change of position or fracture of their fixation material. The pathological radiological findings were not clinically relevant in any of the patients.

## Discussion

In this consecutive series of 303 patients with S-CS-fx treated with open surgical fixation, the median age was 48 years, and 74 % were males. At the time of surgery, 43 % had a SCI. Radiculopathy, which was only registered in cases without SCI, was diagnosed in 27 % of the patients. Thus, 70 % of the patients had either SCI or radiculopathy at the time of surgery.

The surgical approach was anterior, posterior, and combined in 69, 22 and 9 % of the patients, respectively. The risk of SCI deterioration or new-onset radiculopathy related to surgery was 2.0 and 1.3 %, respectively. The surgical mortality was 2.3 %. A second surgery, for all indications, was necessary in 7.3 % of the patients. At the long-term follow-up, 51 % of the patients with SCI had improved one or more AIS grades, and preoperative radiculopathy had resolved in 89 % of the patients. Stable fusion was achieved in 98 % of the patients.

### Selection for surgery

Patient selection for surgical treatment may be challenging and will be relatively dependent on the treating surgeon’s considerations. In recent years, we have used the SLIC recommendations for guidance [3]. In our surgical series, 92.4 % had SLIC scores ≥4 points prior to surgery, which was consistent with the recommendation for surgery. The remaining 7.6 % patients had SLIC scores <4 points, which indicates non-surgical treatment. Overall, the compliance to the SLIC recommendations was high in our series.

### Timing of the surgery

Many studies have addressed the timing of surgery in the setting of SCI and spinal cord compression [14]. In recent years, the most commonly used distinction between early and late surgery has been before or after 24 h following trauma. An increasing number of publications have shown the potential benefit of early decompression with respect to neurological recovery [6, 7, 10]. Additionally, patients with spinal fracture without SCI may benefit from early surgery in terms of earlier mobilization, reduced length of hospitalization and a decreased complication rate [12]. A significant proportion of our CS-fx patients were not eligible for early surgery because of logistical/transport challenges, other life-threatening injuries demanding treatment priority, or delayed diagnosis. In some cases where the surgical indication was not clear, we used additional assessments over days prior to the final decision-making. Furthermore, in some patients with CS-fx without any medullary compression, we might consider surgery to obtain the best long-term outcome, even though the mechanical instability may not be immediately threatening. These patients will often be mobilized with a brace pending fixation surgery, and in these cases, we usually consider the timing of the surgery within a few days to be adequate. Among patients for whom surgery was the primary intended treatment, 26 % were operated within 24h, and 65 % within three days. However, the timing range was relatively large among the remaining patients, with the most delayed surgery occurring at 80 days due to a correspondingly delayed diagnosis. In our group of patients who were first selected for non-surgical treatment and then reassigned to surgery due to treatment failure, the timing range was even more expansive. This finding reflects that the reassignment to surgery was based on a large variety of indications, from acute secondary dislocation with the onset of SCI to slowly developing kyphosis without neurological impact. Patients who were converted from conservative treatment to surgery will be studied in detail in a separate study.

### SCI deterioration related to surgery

The reported rate of SCI in CS-fx depends on the patient material presented, with a higher incidence of SCI in subaxial versus high cervical fractures (C0–C2), in surgically versus conservatively treated fractures, and in selected trauma subgroups versus all CS-fx in a general population [2].

Many studies have reported no neurological worsening after fixation surgery for S-CS-fx, all of which reported a limited number of patients. Kasimatis et al. published an overview of these studies in 2009 [8]. Two studies reported neurological deterioration related to surgery in 2.4–5.2 % of the patients, which is comparable to our deterioration rate of 2.0 % [7, 22]. In our series, five of the six patients who deteriorated had a SCI before surgery. In four patients, the worsened SCI was temporary. Attempts to prevent such deterioration are of major importance. Factors to consider include gentle fiberoptic intubation, careful operating table positioning and gentle surgical technique, along with measures to prevent postoperative hematoma. Perioperative neuromonitoring and spinal navigation may be of benefit in selected cases.

### New-onset radiculopathy after surgery

We did not evaluate potential radiculopathy in patients with SCI, as this would have been too ambiguous to extract from the clinical status, particularly from a retrospective chart review.

The main focus in most studies reporting neurological complications after S-CS-fx surgery is SCI. A review of posterior fusion techniques concluded that the risk of radiculopathy after lateral mass screw fixation was 1 %; however, this study included all indications, not only trauma [23]. A limited series using lateral mass screw fixation for S-CS-fx reported a 2.9 % risk of nerve root injury [24]. Our rate of radicular complications related to surgery was 1.3 %, which is within the range of complication rates reported by previous studies. However, the basis for comparison is limited, and most available studies used posterior fusion techniques to obtain this outcome. In our patient group, three of the four patients who experienced new-onset radiculopathy after surgery were operated using anterior approach.

### Complications requiring secondary surgery

#### Infection

Other studies have reported complication rates for deep wound infection/ resurgery for infection in 1.3–8.8 % of cases, which were mainly related to a posterior approach [7, 9, 13, 24]. Four (1.3 %) of our patients required resurgery for this indication, all of whom were operated using the posterior approach (in two patients, a combined 360° approach was performed).

#### Fixation device failure/ malalignment/inadequate decompression

In our series, these indications for resurgery involved 5.0 % of the patients. Some reports have used similar terms, while other studies have used “construct failure requiring resurgery,” which had a reported frequency of 3.1–4.1 % [4, 7, 11].

#### CSF leakage

Two patients (0.7 %) required resurgery due to CSF leakage. In both patients, the dural tear was caused by bony fragments from the initial trauma. The dural tears were tentatively managed during the first surgery, albeit unsuccessfully. In 2008, Lambiris et al. reported a 0.9 % dural injury rate from surgery for CS-fx, but they did not specify whether this resulted in reoperation [11]. Luszczyk et al. reported a 9.1 % rate of traumatic dural tears in surgically treated cervical spine injury; however, only 0.3 % of the overall cervical spine injury patient group had complications with persistent CSF leakage after the initial surgery [25].

#### Hematoma

Our reoperation rate for postoperative hematoma was 0.3 %. Other studies have reported postoperative hematoma evacuation involving 0.9–3.1 % of their cases [4, 11].

### Surgical mortality

The surgical mortality (death within 30 days after surgery) was 2.3 % in our series. Five of the seven patients were ≥80 years of age, and all died of respiratory failure. In two of these patients, death was apparently partly caused by the surgery given that they both sustained severe SCI deterioration. The other three patients had respiratory failure caused by multitrauma and predisposing morbidity. Two patients <80 years who expired had severe head injury, and this was the direct cause of death. Other studies have reported surgical mortality rates of 3.8–4.5 % [5, 11]. In the STASCIS study, the surgical mortality rate was as low as 0.6 %; however, they did not include patients older than 80 years of age, patients with head injury (GCS ≤13), or patients with severe concomitant injuries [7]. Had we used the same inclusion criteria, our surgical mortality rate would have been 0 %.

### Fusion rate

Cervical spine CTs at the long-term follow-ups revealed 98 % stable fusion. Other studies have reported 88–100 % fusion rates [4, 5, 8, 9, 13, 15]. Achievement of final stability is the primary goal for fixation surgery, and this result is therefore considered very adequate.

### Hoarseness

At the long-term follow-up, 6 % of our patients complained of self-reported hoarseness. Voice-related disorders are well-known complications due to the anterior cervical approach, particularly in the early postoperative weeks [26]. Subjective hoarseness present at long-term after cervical spine surgery may be related to both direct injury to the recurrent laryngeal nerve and other multifactorial causes (e.g., intubation, multisegment fusion, SCI, predisposing factors). The majority of articles addressing subjective voice problems after cervical spine surgery have examined patients undergoing an elective anterior approach for a degenerative disease. Several studies have reported no sustained voice disorders at the long-term follow-up, whereas other studies have reported a frequency of 9–19 % [27–29]. The incidence of persistent voice disorders is higher for multilevel fusion and surgery above the C4 level [28, 29]. In our patient group, this complaint was equally frequent in patients who were operated with posterior as with anterior approaches, indicating multifactorial etiologies.

### Dysphagia

Dysphagia is a subjective symptom. It is a well-known early complication of anterior cervical spine surgery [27, 30]. However, persistent dysphagia has been shown to be as pronounced after anterior approaches as after posterior cervical spinal approaches, and also when comparing ACDF with posterior lumbar surgery [31, 32]. Radcliff et al. observed dysphagia at baseline (prior to surgery) in 11 % in their patients [31]. The incidence of persistent dysphagia after anterior surgical approach to the cervical spine was reported to range from 0 to 28 % [27, 28, 30].

Difficulties with swallowing to some degree was self-reported by 9 % of our patients at follow-up. This finding was not significantly related to any of the surgical approaches in particular.

### Sustained neck pain

At follow-up, the mean VAS score for neck pain was 1.9. Additionally, 80 % of the patients had VAS scores ≤3, and 5 % had VAS scores ≥7. Thus, the majority did not experience significant neck pain as a chronic problem after surgically treated S-CS-fx. Reindl et al. reported that 12.2 % of their patients treated with anterior approach for S-CS-fx had neck pain with VAS ≥7 at follow-up [22]. Brodke et al. found that 29.8 % of their patients treated with anterior or posterior approach for S-CS-fx reported sustained neck pain at follow-up; however, the pain severity was not further clarified [5].

### Sustained neck stiffness

At follow-up, 11 % of our patients reported severe neck stiffness, and this was significantly related to the posterior approach. This finding was expected considering the direct mechanical consequences when fusion of four subaxial segments is a standard procedure, combined with the long muscular incision. A cadaver study demonstrated that the mechanical cervical spine stiffness is greater after posterior fusion techniques compared with ACDF and that the lateral mass screw produced greater stiffness than wiring techniques [33].

### Long-term neurological outcomes

#### SCI

In the large prospective cohort multicenter study (STASCIS), the investigators compared neurological outcomes after surgical decompression before and after 24 h for acute cervical SCI [7]. Neurological outcomes were registered at the 6 month follow-up. In their early (<24 h) group, 56.5 % of the patients had at least one AIS grade improvement, whereas 19.8 % improved ≥2 AIS grades. One patient (0.8 %) experienced AIS grade worsening. In the late group (≥24 h), AIS grade improvement of one or more was observed in 49.5 % of patients, and 8.8 % of patients had improvement of ≥2 grades.

At the follow-up in our population, no patient had an inferior AIS grade compared with the preoperative status. Of all the patients with SCI available for long-term follow-up, 51.4 % had an improvement in AIS grades, whereas 15.5 % improved ≥2 AIS grades. As in STACSIS, we also observed a trend that more patients gained a considerable improvement in AIS (≥2 grades) in the early versus the late surgery group; however, in our series, this difference was not significant. In the STASCIS study, patients with head trauma (GCS ≤13), severe concomitant injuries, and age >80 years were excluded. In our series, these patients were not excluded. Thus, the two series are not directly comparable.

#### Radiculopathy

At presentation, radiculopathy was the only neurological symptom in 27 % of the patients. The rate of new-onset radiculopathy in relation to the surgery was 1.3 %. The outcome with respect to radiculopathy at the time of follow-up was very good. The radiculopathy completely resolved in 89 % of patients. Ten patients had a radiculopathy at follow-up, nine of whom had had a radiculopathy at the time of surgery, and one patient had new-onset radiculopathy after surgery. In all patients with persistent radiculopathy, the radicular complaints were milder than those experienced preoperatively. Woodworth et al. reported a complete resolution of radiculopathy in 91 % of patients at follow-up after surgically treated S-CS-fx [15].

### Limitations of the study

The retrospective data collection is a major limitation.

S-CS-fx encompass a broad spectrum of different injury patterns. Patient selection for surgical treatment may be demanding, with reasonable expectations that this selection might vary between decision makers. Additionally, the selection of surgical techniques for the same types of injuries will differ between surgeons. These factors may limit the external validity of this study. We do not have the records of all of the patients treated non-surgically for S-CS-fx within the same time-frame. This limits the evaluation of our practice in conjunction with the guidelines and restricts our possibilities to compare outcomes with non-surgically treated patients.

### Strengths of the study

- This is a large contemporary and consecutive series of surgically treated patients with S-CS-fx.
- The patient population is well defined within a geographic region, with one neurosurgical center executing all surgery for CS-fx within this population. This reduces the bias with respect to age, socioeconomic status, trauma mechanism, trauma severity, multitrauma and comorbidity.
- The follow-up component of the study was performed prospectively, and the participation was excellent, in which 99.2 % of the surviving patients living in Norway fulfilled the CT and clinical analyses.

## Conclusions

Subaxial cervical spine fractures encompass a broad spectrum of injuries, from less severe injuries for which non-surgical treatment may be employed successfully to life-threatening injuries, which confer a high risk of morbidity. Patient selection for surgical treatment may be demanding, and thus it is of major importance to understand the risks of surgery and the long-term outcomes after surgery.

In this large consecutive series of patients with S-CS-fx treated with open surgical fixation, the surgical mortality was 2.3 %, the risk of neurological deterioration was 3.3 %, and the reoperation rate (any cause) was 7.3 %. The long-term results regarding neurologic function were good, with AIS grade improvement in 51 % and resolution of radiculopathy in 89 % of the patients. Stable fusion was excellent and was achieved in 98 % of cases. Considering the high risk of morbidity that subaxial cervical spine fractures may entail, the surgical risk in this series is considered acceptable.

## Abbreviations

ACDF, anterior cervical decompression and fusion; AIS, American Spinal Injury Association impairment scale; CS-fx, cervical spine fracture(s); HISS, head injury severity scale; SCI, spinal cord injury; S-CS-fx, subaxial cervical spine fracture(s); SLIC, the subaxial injury classification system; VAS, the visual analog scale

## References

[1] Fredø HL, Bakken IJ, Lied B, Rønning P, Helseth E (2014). Incidence of traumatic cervical spine fractures in the Norwegian population: a national registry study. Scand J Trauma Resusc Emerg Med.

[2] Fredø HL, Rizvi SA, Lied B, Rønning P, Helseth E (2012). The epidemiology of traumatic cervical spine fractures: a prospective population study from Norway. Scand J Trauma Resusc Emerg Med.

[3] Vaccaro AR, Hulbert RJ, Patel AA, Fisher C, Dvorak M, Lehman RA, Anderson P, Harrop J, Oner FC, Arnold P (2007). The subaxial cervical spine injury classification system: a novel approach to recognize the importance of morphology, neurology, and integrity of the disco-ligamentous complex. Spine.

[4] Belirgen M, Dlouhy BJ, Grossbach AJ, Torner JC, Hitchon PW (2013). Surgical options in the treatment of subaxial cervical fractures: a retrospective cohort study. Clin Neurol Neurosurg.

[5] Brodke DS, Anderson PA, Newell DW, Grady MS, Chapman JR (2003). Comparison of anterior and posterior approaches in cervical spinal cord injuries. J Spinal Disord Tech.

[6] Dvorak MF, Noonan VK, Fallah N, Fisher CG, Finkelstein J, Kwon BK, Rivers CS, Ahn H, Paquet J, Tsai EC (2015). The influence of time from injury to surgery on motor recovery and length of hospital stay in acute traumatic spinal cord injury: an observational Canadian cohort study. J Neurotraum.

[7] Fehlings MG, Vaccaro A, Wilson JR, Singh A, WC D, Harrop JS, Aarabi B, Shaffrey C, Dvorak M, Fisher C (2012). Early versus delayed decompression for traumatic cervical spinal cord injury: results of the Surgical Timing in Acute Spinal Cord Injury Study (STASCIS). PLoS One.

[8] Kasimatis GB, Panagiotopoulos E, Gliatis J, Tyllianakis M, Zouboulis P, Lambiris E (2009). Complications of anterior surgery in cervical spine trauma: an overview. Clin Neurol Neurosurg.

[9] Kwon BK, Fisher CG, Boyd MC, Cobb J, Jebson H, Noonan V, Wing P, Dvorak MF (2007). A prospective randomized controlled trial of anterior compared with posterior stabilization for unilateral facet injuries of the cervical spine. J Neurosurg Spine.

[10] La Rosa G, Conti A, Cardali S, Cacciola F, Tomasello F (2004). Does early decompression improve neurological outcome of spinal cord injured patients? Appraisal of the literature using a meta-analytical approach. Spinal Cord.

[11] Lambiris E, Kasimatis GB, Tyllianakis M, Zouboulis P, Panagiotopoulos E (2008). Treatment of unstable lower cervical spine injuries by anterior instrumented fusion alone. J Spinal Disord Tech.

[12] Pakzad H, Roffey DM, Knight H, Dagenais S, Yelle JD, Wai EK (2011). Delay in operative stabilization of spine fractures in multitrauma patients without neurologic injuries: effects on outcomes. Can J Surg.

[13] Song KJ, Lee KB (2008). Anterior versus combined anterior and posterior fixation/fusion in the treatment of distraction-flexion injury in the lower cervical spine. J Clin Neurosci.

[14] van Middendorp JJ, Hosman AJ, Doi SA (2013). The effects of the timing of spinal surgery after traumatic spinal cord injury: a systematic review and meta-analysis. J Neurotraum.

[15] Woodworth RS, Molinari WJ, Brandenstein D, Gruhn W, Molinari RW (2009). Anterior cervical discectomy and fusion with structural allograft and plates for the treatment of unstable posterior cervical spine injuries. J Neurosurg Spine.

[16] Statistisk sentralbyra. [http://www.ssb.no]

[17] WHO (2009). ICD-10: International statistical classification of diseases and related health problems, vol. 10th revision.

[18] Stein SC, Spettell C (1995). The Head Injury Severity Scale (HISS): a practical classification of closed-head injury. Brain Inj.

[19] Kirshblum SC, Waring W, Biering-Sorensen F, Burns SP, Johansen M, Schmidt-Read M, Donovan W, Graves D, Jha A, Jones L (2011). Reference for the 2011 revision of the international standards for neurological classification of spinal cord injury. J Spinal Cord Med.

[20] Huskisson EC (1974). Measurement of pain. Lancet (London, England).

[21] Buuren S, Groothuis-Oudshoorn K (2011). Mice: multivariate imputation by chained equations in R. J Stat Softw.

[22] Reindl R, Ouellet J, Harvey EJ, Berry G, Arlet V (2006). Anterior reduction for cervical spine dislocation. Spine.

[23] Coe JD, Vaccaro AR, Dailey AT, Skolasky RL, Sasso RC, Ludwig SC, Brodt ED, Dettori JR (2013). Lateral mass screw fixation in the cervical spine: a systematic literature review. J Bone Joint Surg Am.

[24] Pateder DB, Carbone JJ (2006). Lateral mass screw fixation for cervical spine trauma: associated complications and efficacy in maintaining alignment. Spine J.

[25] Luszczyk MJ, Blaisdell GY, Wiater BP, Bellabarba C, Chapman JR, Agel JA, Bransford RJ (2014). Traumatic dural tears: what do we know and are they a problem?. Spine J.

[26] Beutler WJ, Sweeney CA, Connolly PJ (2001). Recurrent laryngeal nerve injury with anterior cervical spine surgery risk with laterality of surgical approach. Spine.

[27] Fountas KN, Kapsalaki EZ, Nikolakakos LG, Smisson HF, Johnston KW, Grigorian AA, Lee GP, Robinson JS (2007). Anterior cervical discectomy and fusion associated complications. Spine.

[28] Mehra S, Heineman TE, Cammisa FP, Girardi FP, Sama AA, Kutler DI (2014). Factors predictive of voice and swallowing outcomes after anterior approaches to the cervical spine. Otolaryngol Head Neck Surg.

[29] Yue WM, Brodner W, Highland TR (2005). Persistent swallowing and voice problems after anterior cervical discectomy and fusion with allograft and plating: a 5- to 11-year follow-up study. Eur Spine J.

[30] Cho S, Lu Y, Lee D (2013). Dysphagia following anterior cervical spinal surgery: a systematic review. Bone Joint J.

[31] Radcliff KE, Koyonos L, Clyde C, Sidhu GS, Fickes M, Hilibrand AS, Albert TJ, Vaccaro AR, Rihn JA (2013). What is the incidence of dysphagia after posterior cervical surgery?. Spine.

[32] Siska PA, Ponnappan RK, Hohl JB, Lee JY, Kang JD, Donaldson WF (2011). Dysphagia after anterior cervical spine surgery: a prospective study using the swallowing–quality of life questionnaire and analysis of patient comorbidities. Spine.

[33] Murakami H, Jarrett C, Rhee JM, Tsai L, Hutton W (2010). Spinous process wiring versus lateral mass fixation for the treatment of anterior cervical pseudarthrosis: a biomechanical comparison. J Surg Orthop Adv.

